# Fibromyalgia and non-celiac gluten sensitivity: a description with remission of fibromyalgia

**DOI:** 10.1007/s00296-014-2990-6

**Published:** 2014-04-12

**Authors:** Carlos Isasi, Isabel Colmenero, Fernando Casco, Eva Tejerina, Natalia Fernandez, José I. Serrano-Vela, Maria J. Castro, Luis F. Villa

**Affiliations:** 1 Department of Rheumatology, Hospital Puerta de Hierro, Majadahonda Madrid, Spain; 2 Department of Pathology, Hospital Infantil Niño Jesús, Madrid, Spain; 3 Department of Pathology, Hospital Puerta de Hierro, Majadahonda Madrid, Spain; 4 Department of Gastroenterology, Hospital Puerta de Hierro, Majadahonda Madrid, Spain; 5 Celiac and Gluten Sensitive patients Association of Madrid, Madrid, Spain; 6 Department of Immunology, Hospital Doce de Octubre, Madrid, Spain

**Keywords:** Fibromyalgia, Celiac disease, Gluten sensitivity, Intraepithelial lymphocytosis

## Abstract

Fibromyalgia (FM) syndrome is a disabling clinical condition of unknown cause, and only symptomatic treatment with limited benefit is available. Gluten sensitivity that does not fulfill the diagnostic criteria for celiac disease (CD) is increasingly recognized as a frequent and treatable condition with a wide spectrum of manifestations that overlap with the manifestations of FM, including chronic musculoskeletal pain, asthenia, and irritable bowel syndrome. The aim of this report was to describe 20 selected patients with FM without CD who improved when placed on a gluten-free diet. An anti-transglutaminase assay, duodenal biopsy, and HLA typing were performed in all cases. CD was ruled out by negative anti-transglutaminase assay results and absence of villous atrophy in the duodenal biopsy. All patients had intraepithelial lymphocytosis without villous atrophy. Clinical response was defined as achieving at least one of the following scenarios: remission of FM pain criteria, return to work, return to normal life, or the discontinuation of opioids. The mean follow-up period was 16 months (range 5–31). This observation supports the hypothesis that non-celiac gluten sensitivity may be an underlying cause of FM syndrome.

## Introduction

Fibromyalgia (FM) is a commonly recognized syndrome characterized by pain, sleep disturbance, and fatigue combined with a general increase in medical symptoms, including problems of memory or thinking, and often psychological distress [1]. The pharmacological treatment of FM results in only partial relief [2]. This condition is frequently associated with depression and irritable bowel syndrome.

Celiac disease (CD) is a frequent disease, affecting about 1 % of the population that can be diagnosed at all ages under different clinical settings. The classic presentation of CD is chronic diarrhea, positive anti-tissue transglutaminase antibodies (anti-tTG), and villous atrophy with intraepithelial lymphocytosis observed by duodenal biopsy. CD often does not conform to the classical clinical description, and up to 50 % of patients with adult CD lack prominent gastrointestinal symptoms [3, 4]. Clinical manifestations of CD include muscle and osteoarticular pain, fatigue, and neurological and psychological symptoms [5, 6].

Non-celiac gluten sensitivity is increasingly recognized as a frequent clinical condition with symptoms similar to CD in the absence of the diagnostic features of CD [7, 8]. Intraepithelial lymphocytosis without villous atrophy and without specific celiac antibodies has been shown to be part of the spectrum of non-celiac gluten sensitivity. These mild gluten-related lesion have been given different names, such as gluten-sensitive enteropathy or mild celiac enteropathy [9–12]. The aim of this report is to describe selected patients with FM, non-celiac gluten sensitivity, duodenal intraepithelial lymphocytosis, and clinical response to gluten-free diet, in order to raise the hypothesis that non-celiac gluten sensitivity could be a treatable cause of FM.

## Methods

As part of a wider research project approved by the local ethics committee, patients diagnosed with FM and referred to a widespread chronic pain rheumatology unit were given the option to undergo fibrogastroscopy with duodenal biopsies, despite negative anti-tTG assay results, and gave informed consent for fibrogastroscopy knowing the negative results of the CD serology. After the pathology report ruled out CD, consented to participating in an open gluten-free diet trial. Currently, we are following 246 patients of these patients. Clinical response has been observed in 90 of them. This is a report of the first patients for whom duodenal intraepithelial lymphocytosis and a clinical response to a gluten-free diet were observed. These patients also gave additional consent for their cases to be published, including their genetic data.

Duodenal biopsies were obtained from the second duodenal portion before beginning the GFD. These samples were retrospectively evaluated by three pathologists from two academic centers with anti-CD3 T-cell receptor immunohistochemical stains. The identification of intraepithelial lymphocytosis was agreed upon by the three pathologists and considered to be present when the intraepithelial lymphocyte count was more than 25 per 100 enterocytes [13–15]. HLA-DRB1, HLA-DQA1, and HLA-DQB1 alleles were determined from blood samples.

A trial of a strict gluten-free diet was conducted with the help and training of the CD Patients Association of Madrid. In addition, iron, vitamin D, and multivitamins supplements with oligoelements were prescribed. A lactose-free diet was also recommended when lactose intolerance was suspected on clinical grounds or demonstrated by a breath hydrogen test after lactose administration. Clinical response was defined as the achievement of at least one of the following scenarios: remission of FM pain criteria, return to work, return to normal life as judged by the patient, or opioid discontinuation.

## Results

Twenty patients with FM are described for whom duodenal intraepithelial lymphocytosis and a clinical response to a gluten-free diet were observed. Table 1 shows the clinical and HLA descriptions of each patient. All subjects were female. The mean subject age was 46 years (range 25–73). The mean duration of FM evolution was 11.9 years (range 3–20). Eight patients had been diagnosed with chronic fatigue syndrome. Seventeen patients had a gastrointestinal disorder (irritable bowel syndrome, gastroesophageal reflux, constipation, dyspepsia, hiatal hernia, lactose intolerance, or nonspecific colitis). Other diagnoses were depression (eight patients), migraine (eight patients), and hypothyroidism without improvement in FM with thyroid hormone substitution (three patients). Five patients had features of undifferentiated spondyloarthritis, and one patient had psoriatic arthritis.

**Table 1:** Descriptions of the twenty patients with fibromyalgia and duodenal IEL and their responses to a gluten-free diet

	Previous diagnoses	Age	Years evol	HLA susceptibility	Treatment	GFD months	Description of improvement
1	FM, CFS, migraine, GER, lactose intolerance	49	20	DQ8 DQA1*0301 DQB1*0302 DQ7 DQA1*0505 DQB1*0301	GFD, LFD, vit D, Fe, mv sup	31	Remission of FM Also improved: fatigue, GIs, migraine Limited life, sick leave/Returns to active life and to work
2	FM, migraine, oral aphthae, psoriatic arthritis	35	7	DQ7 DQA1*0303 DQB1*0301 DQ6	GFD, vit D, Fe, mv sup	30	Remission of FM, Remission of psoriatic arthritis and aphthae Limited home life/active life
3	FM, migraine, iron deficiency anemia, oral aphthae, uSpA, depression, hyperprolactinemia	41	15	DQ2 DQA1*0201 DQB1*0202 DQ6	GFD, mv sup	8	Remission of FM. Remission of aphthae Also improved: back pain, fatigue, GIs, migraine, depression, Limited life/normal life
4	FM, CFS, dyspepsia, constipation	61	10	DQ2 DQA1*0501 DQB1*0201 DQ2 DQA1*0201 DQB1*0202	GFD, LFD, mv sup	6	Remission of FM Also improved: fatigue, GIs Improved daily activities
5	FM, depression, hypothyroidism, constipation	46	10	DQ7 DQA1*0505 DQB1*0301 DQ6	GFD, LFD vit D, mv sup	24	Remission of FM Also improved: fatigue, GIs, depression Limited life/returns to work
6	FM, IBS, depression	57	20	DQ2 DQA1*0501 DQB1*0201 DQ7 DQA1*0303 DQB1*0301	GFD, LFD, mv sup	17	Remission of FM Also improved: fatigue, GIs, depression In Pain Unit with opioids, on sick leave/return to work, discontinuation of opioids
7	FM, dyspepsia	73	5	DQ2 DQA1*0501 DQB1*0201 DQ4	GFD	7	Remission of FM Also improved: asthenia, GIs Limited life/normal life
8	FM, IBS, iron deficiency anemia, OP, migraine	61	10	DQ2 DQA1*0501 DQB1*0201 DQ2 DQA1*0501 DQB1*0201	GFD	5	Remission of FM Also improved: fatigue, GIs, migraine
9	FM, GER, migraine, nonspecific colitis, uSpA	42	10	DQ2 DQA1*0201 DQB1*0202 DQ6	GFD	22	Remission of FM Also improved: back pain, fatigue, GIs, migraine Limited life/normal life
10	FM, CFS, depression, hypothyroidism, psoriasis, iron deficiency anemia, dyspepsia	42	8	DQ2 DQA1*0201 DQB1*0202 DQ9	GFD, vit D, Fe, mv sup	18	Improved: pain, fatigue, GIs, depression Sick leave/return to work
11	FM, IBS, uSpA	49	20	DQ2: DQA1*0501 DQB1*0201 DQ6	GFD, vit D, mv sup	24	Improvement in pain, fatigue, and GIs Limited life/normal life
12	FM, CFS depression, gastritis, hiatal hernia	50	20	DQ8: DQA1*0301 DQB1*0302 DQ2: DQA1*0201 DQB1*0202	GFD	5	Improvement in pain, fatigue, and GIs Limited life/return to active life
13	FM, CFS, migraine, obesity, SAHS, constipation, esophagitis	37	7	DQ2 DQA1*0201 DQB1*0202 DQ6	GFD, LFD,	24	Remission of FM Also improved: fatigue, GIs, migraine Sick leave/return to work
14	FM, uSpA, dyspepsia, migraine	49	12	DQ8: DQA1*0301 DQB1*0302	GFD	6	Remission of FM Also improved: back pain, fatigue, GIs, migraine Limited life/normal life
15	FM, dyspepsia	25	5	DQ7 DQA1*0505 DQB1*0301 DQ6	GFD	24	Remission of FM Also improved: fatigue, GIs Limited life/normal life
16	FM, IBS, depression	37	15	DQ9 DQ5	GFD	16	Improvement of pain, fatigue, depression, and GIs Chronic opioids and home limited life/occasional opioid use, goes to gym and swimming
17	FM, CFS	33	3	DQ2 DQA1*0501 DQB1*0201 DQ5	GFD	8	Remission of FM Also improved: fatigue, Limited/normal life
18	FM, CFS, migraine, vitiligo	43	15	DQ8 DQA1*0301 DQB1*0302 DQ2:DQA1*0201 DQB1*0202	GFD	6	Remission of FM Also improved: fatigue, Limited/normal life
19	FM, CFS, IBS, migraine, depression, somatizing disorder, uSpA	42	20	DQ2 DQA1*0201 DQB1*0202 DQ5	GFD, LFD	24	Remission of FM pain Also improved: fatigue, GIs, depression In Pain Unit with opioids, bed limited life/normal active life, discontinuation of opioids
20	FM, hypothyroidism, depression, dyspepsia	61	6	DQ2: DQA1*0501 DQB1*0201 DQ6	GFD, LFD, mv sup	24	Improvement in pain, fatigue, GIs, depression Limited life/normal life

*FM* fibromyalgia, *CFS* chronic fatigue syndrome, *IBS* irritable bowel syndrome, *GER* gastroesophageal reflux, *uSpA* undifferentiated spondyloarthritis, *OP* osteoporosis, *SAHS* sleep apnea hypopnea syndrome, *GFD* gluten-free diet, *LFD* lactose-free diet, *mv sup* multivitamin and oligoelements supplementation, *GIs* gastrointestinal symptoms

Eleven patients carried either the DQ2 (DQA1*05—DQB1*02) or DQ8 (DQA1*0301–DQB1*0302) heterodimers. Seven patients had only one allele of the DQ2 heterodimer, either DQB1*02 or DQA1*05. Two patients did not carry either DQ2 alleles or DQ8.

The mean follow-up period for the gluten-free diet was 16.4 months (range 5–31). Eight patients were also on a lactose-free diet. For five of these eight patients, a lactose-free diet had been attempted before the gluten-free diet, resulting in partial relief of the gastrointestinal symptoms but no improvement in the FM symptoms. For three patients (#4, 10, and 20), a lactose-free diet was started concurrently with the gluten-free diet.

The level of widespread chronic pain improved dramatically for all patients; for 15 patients, chronic widespread pain was no longer present, indicating remission of FM. Fifteen patients returned to work or normal life. In three patients who had been previously treated in pain units with opioids, these drugs were discontinued. Fatigue, gastrointestinal symptoms, migraine, and depression also improved together with pain. Patients #2 and #3, both with oral aphthae, went into complete remission for psoriatic arthritis and undifferentiated spondyloarthritis.

For some patients, the clinical improvement after starting the gluten-free diet was striking and observed after only a few months; for other patients, improvement was very slow and was gradually observed over many months of follow-up. For eight patients (# 2, 3, 8, 9, 12, 14, 17, 19, and 20), the intake of gluten was followed by clinical worsening, which subsided after returning to a strict gluten-free diet.

## Discussion

Here, we report the cases of patients with severe longstanding FM in whom CD was ruled out, but had duodenal intraepithelial lymphocytosis and exhibited clinical responses to a gluten-free diet. The observation for FM patients of a histopathologic lesion associated with a treatable disease opens new perspectives.

The concept of non-celiac gluten sensitivity arises from clinical observations of patients whose symptoms clearly improve or resolve with a gluten-free diet even though CD has been ruled out. Non-celiac gluten sensitivity is currently included in the wider concept of gluten-related disorders, along with CD and wheat allergy [16].

**Fig. 1:**
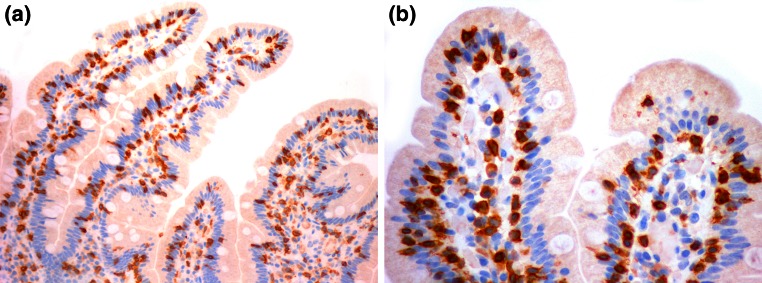
**a** CD3 staining of intraepithelial lymphocytes in the duodenal villi of patient #8. **b** CD3 staining of intraepithelial lymphocytes in the tips of the duodenal villi of patient #1

In non-celiac gluten sensitivity, specific autoantibodies are not present, and duodenal biopsy only reveals intraepithelial lymphocytosis or no pathologic changes. Only approximately half of patients with GS carry the HLA DQ2 or DQ8 heterodimers, in contrast to CD in which almost all patients carry the HLA DQ2 or DQ8. The symptoms of non-celiac gluten sensitivity include behavioral changes, bone or joint pain, muscle cramps, leg numbness, weight loss, chronic fatigue, and a foggy mind. Oral aphthae and psoriasis can also be due to non-celiac gluten sensitivity [7]. Intraepithelial lymphocytosis has been the hallmark of gluten intestinal lesions since the first descriptions of the intestinal pathology of CD [17]. In the current classification criteria, a Marsh 1-type lesion is defined as the presence of more than 25 intraepithelial lymphocytes per one hundred enterocytes in a duodenal biopsy. Intraepithelial lymphocytosis can be overlooked or not considered relevant in a pathology report focused on villous atrophy or can be missed if anti-CD3 staining is not performed [13, 14]. Although intraepithelial lymphocytosis is associated with several other conditions, such as *Helicobacter pylori* disease, NSAID intake, Crohn’s disease, or parasitic infestation, gluten enteropathy must always be considered [18–20]. For our patients, intraepithelial lymphocytosis was found by experienced pathologists after a specific search. Two examples of intraepithelial lymphocytosis are shown in Fig. 1, which presents images of CD3-stained preparations. The high frequency of HLA alleles related to susceptibility to CD in our patients is similar to that described in non-celiac gluten sensitivity.

The spectrum of symptoms of our patients is wide and complex, as is typical for FM and described in non-celiac gluten sensitivity. The reduction in the level of pain was accompanied by improvements in asthenia and gastrointestinal and neurological symptoms, suggesting a common underlying cause related to gluten. The clinical response definition for this report was decided upon after the initial observations of impressive improvement in some patients after starting the gluten-free diet. We chose demanding outcome measures, such as the remission of FM or return to normal life, rather than changes reflected by questionnaires. To our knowledge, this degree of improvement has not been reported previously for FM. It is unlikely that our results were caused by a placebo effect. The majority of patients had severe distress and disability and were unable to cope normally with daily activities or on sick leave, despite having been treated with many different regimens for years. In FM clinical trials, the placebo effect has been very modest and transient, in contrast to the great long-term improvement of our patients. Lactose-free diet and the addition of vitamins could be confounders, but the majority of the patients with lactose-free diet had tried it before without improvement of FM, and in our experience FM does not have relevant clinical improvement just adding vitamins and minerals. Furthermore, the reintroduction of gluten, for 7 patients, was followed by FM relapse; when re-introduced, the strict gluten-free diet led to clinical improvement.

This report describes the first patients for which we have observed FM with intraepithelial lymphocytosis and the response to a gluten-free diet. Currently, we are following 246 patients with FM on a strict gluten-free diet, and the degree of clinical improvement described in this report has been observed in 90 of them. We still do not have long-term follow-up and CD3 staining data for all of our patients. However, it is worth communicating our first observations, which open a wide field for research. Indeed, non-celiac gluten sensitivity as a cause of FM, and the role of duodenal intraepithelial lymphocytosis, needs to be tested in a controlled trial.

## Conclusion

This report shows that remarkable clinical improvement can be achieved with a gluten-free diet in patients with FM, even if CD has been ruled out, suggesting that non-celiac gluten sensitivity may be an underlying treatable cause of FM syndrome. The presence of intraepithelial lymphocytosis in the duodenal biopsies of these selected patients further supports this hypothesis.
